# 2024 Aspen Healthcare & Quality of Life Seminar: forging a blueprint for Romania's healthcare transformation

**DOI:** 10.25122/jml-2024-1006

**Published:** 2024-04

**Authors:** Teodor Blidaru, Carmen-Mihaela Bardoș, Iulia Arif-Perca, Sebastian Bereș, Ioana Bianchi, Adrian Brîndușan, Anca Bundoi, Codruț Codreanu, Ovidiu-Simion Cotoi, Adrian-Marius Dobre, Loredana Drăgoi, Paul Dudău, Florentina Furtunescu, Andreea Grigore, Thomas Hofmarcher, Anca Ifrim, Grațiela Iordache, Attila László, Delia Lupescu, Cristina Maxim, Larisa Mezinu-Bălan, Richard Mihalache, Vlad Nerău, Bogdan Pană, Cătălin Radu, Raluca Sîmbotin, Iulia Stoea, Ștefan Strilciuc, George Ștefan, Oana Talpoș, Iulian Trandafir, Mircea Vâlceanu, Laurențiu Dașcă

**Affiliations:** 1 Carol Davila University of Medicine and Pharmacy, Bucharest, Romania; 2 Ministry of Health, Bucharest, Romania; 3 Local American Working Group Association (LAWG), Bucharest, Romania; 4 Nordpharm, Bucharest, Romania; 5 The Romanian Association of International Medicine Manufacturers (ARPIM), Bucharest, Romania; 6 Prof. Dr. Ion Chiricuță Oncology Institute, Cluj-Napoca, Romania; 7 Astra Zeneca, Bucharest, Romania; 8 National Health Insurance House, Bucharest, Romania; 9 George Emil Palade University of Medicine, Pharmacy, Science and Technology, Targu Mures, Romania; 10 Unifarm National Company, Bucharest, Romania; 11 Roche, Bucharest, Romania; 12 Medtronic, Bucharest, Romania; 13 AbbVie, Bucharest, Romania; 14 The Swedish Institute for Health Economics, Lund, Sweden; 15 OMNIASIG Vienna Insurance Group, Bucharest, Romania; 16 National Authority for Quality Management in Healthcare of Romania, Bucharest, Romania; 17 Senate representative, Parliament of Romania, Bucharest, Romania; 18 Romanian Presidential Administration, Bucharest, Romania; 19 Ministry of Finance, Bucharest, Romania; 20 Napofarm Pharmacies, Bucharest, Romania; 21 Bucharest University of Economic Studies, Bucharest, Romania; 22 Bristol Myers Squibb Romania; 23 Merck Sharp & Dohme Romania; 24 Research Center for Functional Genomics, Biomedicine and Translational Medicine, Iuliu Hațieganu University of Medicine and Pharmacy, Cluj-Napoca, Romania; 25 Alliance Healthcare, Bucharest, Romania; 26 Arensia Exploratory Medicine, Bucharest, Romania

## INTRODUCTION

Even after COVID-19 transitioned to an endemic phase, healthcare remains a pressing concern for governments. Public and private healthcare spending faces unprecedented pressure due to several factors, including an aging population, increasing migration of active population, and massive investment in research and development of medical innovation [1]. Consequently, evaluating the economic benefits generated by investments in public health systems becomes crucial for assessing current policies and informing future, evidence-based decision-making [2].

Romania's healthcare system faces several critical challenges, as outlined in the Organisation for Economic Co-operation and Development (OECD) country profile [3]. Key issues include inadequate funding, leading to a scarcity of medical personnel and a deterioration in service quality. This has resulted in diminished healthcare access, especially in rural and remote areas [4]. The system also struggles with the migration of healthcare professionals seeking better opportunities abroad, further straining the capacity to deliver quality medical services. Additionally, Romania experiences challenges related to aging demographics, requiring more effective drugs and advanced technology to cope with the healthcare needs of an older population [5].

**Figure 1:**
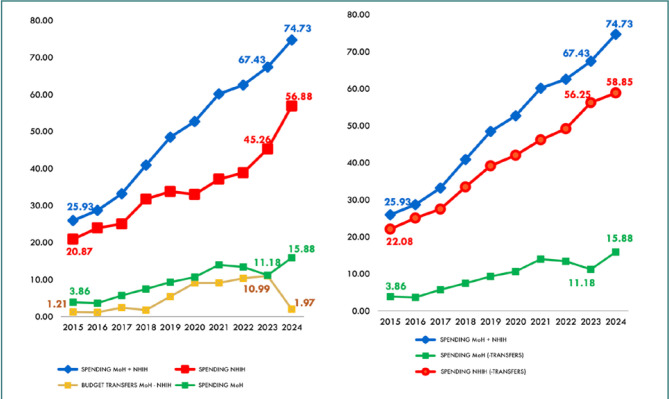
Public healthcare spending by source (Ministry of Health [MoH]/National Health Insurance House [NHIH], RON billions)

Despite significant increases in Romania’s public healthcare spending (Figure 1), the health system remains under strain, facing underfunding, workforce shortages and maldistribution, and technological challenges in meeting the needs of its aging population. Furthermore, its financing is highly fragmented and uneven across similar healthcare institutions [6]. The economic principle of allocating limited resources to meet seemingly unlimited needs is particularly challenging in healthcare, where, despite considerable progress, the resources remain insufficient, and the demands continue to escalate.

**Figure 2:**
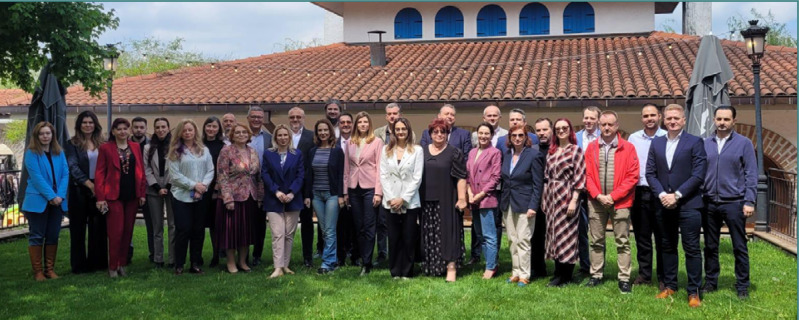
The 2024 Aspen Custom Healthcare Seminar participants, Comana, Giurgiu, Romania

The Aspen Custom Healthcare Seminar, which convened in Comana, Romania (April 25–27, 2024), provided a platform for frank discussion about the urgent challenges and promising opportunities within Romania's healthcare system. The unique gathering included high-level government representatives, influential industry figures, a diverse pool of healthcare professionals, and leading academics (Figure 2).

The seminar delved into the economic impact of public health investments in Romania, examining microeconomic (individual/patient) and macroeconomic (societal) effects. Three potential long-term scenarios were explored: (1) maintaining current investment levels with a focus on optimizing existing spending, (2) increasing investments in prevention and early detection, and (3) allocating additional funds for public health expenditure, including infrastructure and technology.

The discussion centered on key challenges and the impact of various policies and investments on health outcomes. Participants sought to identify best practices for the Romanian healthcare system, including priorities in financing primary and secondary care, major national health programs, and hospital care (using the CaPeSSCoSt project as a case study). The seminar also explored strategies for improving the quality and performance of hospital services through cost evaluation, standardization, and the adoption of successful cost-reduction and efficiency models from other European healthcare systems.

## THE DATA IMPERATIVE

A central theme of the event was the crippling “data blindness” afflicting Romania's healthcare system. Participants lamented the lack of reliable data across numerous critical indicators, ranging from disease prevalence and treatment outcomes to healthcare spending effectiveness. This deficiency inhibits informed decision-making at every level, from resource allocation within hospitals to national-level health policy formulation.

The seminar showcased specific gaps in data collection and analysis. For instance, experts highlighted a lack of granular data on healthcare disparities between rural and urban areas, impeding the design of targeted interventions. Furthermore, underdeveloped systems for tracking patient outcomes over time hinder the ability to evaluate unmet medical needs and treatment effectiveness and identify areas for quality improvement and faster access to innovation. The unanimous verdict: Romania cannot chart a path to a more efficient, equitable healthcare system without first investing heavily in building a robust data infrastructure and dedicated analytical teams. This direction is also boosted by the necessity to converge to the European Health Data Space, aiming to offer patients digital access to personal health records but also make better use of existing health data to improve healthcare outcomes and incentivize research.

## MORE MONEY OR SMARTER SPENDING?

The focus of the seminar on financing priorities underscored the broad consensus: the chronically underfunded healthcare system in Romania desperately needs substantial additional investment to meet rising demands and close the gap with peer nations in the European Union. Nevertheless, the debate intensified over how best to utilize these increased funds. The consensus suggested that the optimal approach should involve a balance between injecting more funds and enhancing efficiency, which could boost public trust and potentially unlock further investments.

The discussion also triggered an overview of the indirect costs for the health system, the society, and the national economy generated by the inefficiencies in the system: lost productivity costs resulting from the need for medical leaves or home care. Another important topic debated in Comana was whether Romania should exclusively invest in its existing public system or whether the introduction of complementary private health insurance schemes could play a beneficial role by increasing the financial protection of Romania’s citizens against catastrophic healthcare spending. Proponents of a purely public model cited potential risks of exacerbating inequities and fragmenting the system. In contrast, advocates for a mixed model argued that private insurance, if carefully regulated, could offer greater choice, reduce pressure on public services, and spur innovation. Furthermore, the development of robust private health services could position Romania as a hub for health tourism, benefiting patients, reducing costs for the national health system, enhancing healthcare workforce retention, and generating substantial revenue for the national economy.

## PREVENTION: THE KEY TO A SUSTAINABLE FUTURE

**Figure 3:**
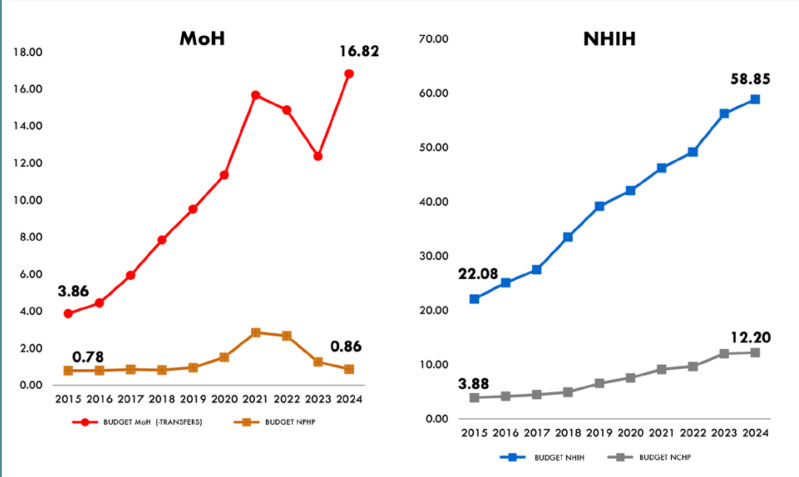
MoH Public Health Programs (left, stationary trend except for the COVID-19 pandemic period) and NHIH Curative Health Programs (left, growth trend) against NHIH and MoH funding (RON billions). *NPHP, National Public Health Programs, NCHP, National Curative Health Programs

Sessions dedicated to primary and secondary care forcefully articulated the need to reorient Romania's healthcare approach towards prevention. Guest speakers, equipped with data showcasing Romania’s lack of funding for prevention (Figure 3) and case studies, demonstrated that investing in preventative services, early disease detection, and chronic disease management yields better population-level health outcomes with reduced overall costs over time. Shifting away from the current hospital-centric model towards a strengthened network of primary care providers was identified as paramount for reducing the future burden on hospitals and creating a healthier Romanian society.

## EQUITY AMONG HEALTHCARE FACILITIES

The issue of inequities within Romania's healthcare system was a central theme of the seminar. Participants debated the causes and consequences of uneven resource allocation across hospitals. Underfunded facilities in certain regions and a lack of specialized care outside major urban centers limit access for many Romanians, disproportionately affecting those with lower socioeconomic status. Seminar discussions centered on devising fairer funding mechanisms prioritizing equity, ensuring that all Romanians – regardless of zip code or income level – have access to high-quality essential healthcare services. Ultimately, sufficient funding is not just a matter of expanding services but a critical factor in safeguarding the well-being and lives of patients.

## ACCESS TO INNOVATION

Experts at the seminar showcased the sluggish pace at which medical innovations reach Romanian patients. The primary barriers identified were insufficient budgets, complex regulatory hurdles, and a lack of robust collaboration between the public and private sectors. The seminar explored potential solutions such as streamlined regulatory pathways, increased predictability and clear timelines throughout the reimbursement process, public-private partnerships dedicated to research and development, and creative financing models to help bring cutting-edge therapies (such as the Health Innovation Program), diagnostics, and medical devices to Romania more rapidly.

Discussions further addressed, more broadly, the cost of underinvesting in healthcare and research & development. The seminar aimed to provide participants with a better understanding of the potential benefits of increased funding for the healthcare system, not merely individual patient benefits but also macro, societal benefits such as long-term cost reduction, increased productivity of the workforce, focusing on the multiplier effect healthcare spending has on the economy.

## PUBLIC-PRIVATE PARTNERSHIPS IN EDUCATION

Public-private partnerships (PPPs) in education play a crucial role in bridging the gap between academic institutions and industry needs. By fostering a collaborative relationship, partners can offer valuable feedback on curriculum development and skills requirements, ensuring educational programs align with current and future job market demands. This partnership allows academia to tailor its educational programs to better prepare students for their careers, making education more relevant and applicable. Additionally, involving future employers and beneficiaries in the educational process helps continuously improve standards and adapt to evolving industry trends.

## INTEGRATING HUMAN RESOURCES AND INFRASTRUCTURE FOR ENHANCED HEALTHCARE DELIVERY

Developing human resources for health is essential, especially when paralleled with investments in infrastructure and services, to fully operationalize and maximize the capabilities of the healthcare system. Adequate training and staffing ensure that new and improved facilities are used effectively and services are delivered efficiently. Additionally, expanding the roles of various health professionals, such as pharmacists and primary healthcare (PHC) doctors and nurses, in preventive care while integrating new/AI-based technologies in healthcare practices can significantly enhance health outcomes. Romania is crossing a period with unprecedented investments in healthcare infrastructure; therefore, this comprehensive approach ensures that these investments are complemented by a skilled workforce capable of delivering high-quality care and preventative services.

## NEXT STEPS: FROM DEBATE TO ACTION

While the Aspen Seminar provided a valuable forum for diagnosing the main healthcare challenges in Romania, it is now imperative to translate these discussions into tangible policy actions.

Key recommendations based on the consensus of authors include:

### 1. The need to invest in **data infrastructure**

Authorities, data scientists, and healthcare providers should focus on developing a comprehensive national strategy for data collection, management, and analysis. This strategy should include investing in electronic health records (EHRs) and interoperable data systems, monitoring access to innovation and the quality of medical services, and training healthcare workers in data literacy and reporting. Additionally, it should regulate and develop the practice of using periodic reports generated from these databases to inform evidence-based political and policy decisions. Such a comprehensive approach will ensure effective data utilization and improve healthcare outcomes.

### 2. Predictability of funding and sustainability of the Romanian health system

The Romanian health system is financed from four main sources: the national health insurance fund, the state and local budgets, and out-of-pocket payments. As the national health insurance fund is responsible for financing universal healthcare coverage for all citizens, it is crucial to explore national strategies for increasing its funding. One effective approach would be reconsidering payment exceptions, ensuring that all individuals and entities contribute fairly to the system. Additionally, expanding the base of contributors can significantly enhance the financial health of the fund. This involves addressing loopholes in the system, clarifying the status of those who currently benefit from healthcare services without contributing and defining the minimal basket of services for the uninsured. By broadening the contribution pool through these measures, the national health insurance fund can secure more consistent and substantial revenue, essential for maintaining and improving the quality of healthcare services across the country. Furthermore, reinvesting revenue from taxes on health-affecting products into the healthcare system to enhance services and promote public health initiatives, especially prevention, should be explored.

Addressing the cash-flow issue of the national health insurance fund should also be a priority and could contribute to the critical need to ensure predictability. The fund is financed from the state budget, with resources provided primarily by contributors (individual and economic entities) and supplemented with subsidies from the state budget and the Ministry of Health. However, ensuring adequate quarterly funding remains a significant challenge due to the differences in spending needs versus the budget transferred, which depends on collections from contributors – with a disproportionate rate of collection compared to the pace at which the budget must be allocated – along with prioritization for supplementing the fund up against other pressing needs across other sectors of the economy. Monthly financial allocation caps are an additional barrier in budget planning and execution, leading to delays in patient access.

### 3. Invest in hospital infrastructure for enhanced healthcare delivery

The infrastructure of public hospitals is in great need of modernization. Upgrading these facilities would improve working conditions for medical personnel and enhance the standard of care provided to patients. Therefore, it is imperative to continue expanding investments in this area while actively seeking new funding sources.

### 4. The health system and its actors should embrace **value-based spending**

Funders should initiate pilot programs that incentivize healthcare providers and hospitals based on patient outcomes rather than solely the volume of services delivered. Value-based models promote efficiency, align spending with results, and encourage a focus on preventive care.

### 5. Championing **transparency and accountability**

Authorities should introduce mechanisms for greater public transparency regarding healthcare spending, performance metrics, and patient outcomes. This can empower citizens and build trust in the system. Regularly publishing data-backed reports on the state of healthcare in Romania would further enhance transparency and facilitate the identification of areas that require improvement.

### 6. Experimenting with **complementary private funding**

The government should consider pilot programs that introduce complementary private insurance options. Such models, if well-regulated, have the potential to alleviate strain on the public system, offer expanded choices, and reduce waiting times for certain services. Robust oversight and clear safeguards are crucial to ensure a mixed-funding approach that does not exacerbate existing inequities.

### 7. Invest in national **healthcare innovation hubs**

Investing in a government-supported entity that fosters collaboration between academia, healthcare providers, and industry could streamline regulatory processes, coordinate clinical research, and attract investment to accelerate the adoption of promising new treatments and technologies throughout Romania. This centralized hub could serve as a catalyst for innovation, driving the development and implementation of effective healthcare solutions. Additionally, the focus should be on promoting and scaling up existing public-private partnerships (PPPs), drawing on successful models from other EU countries to enhance their efficacy and impact locally in improving healthcare outcomes.

### 8. **Clinical trials** and investment in pharmaceutical innovation as an opportunity

Leveraging clinical trials can serve as a significant source of additional funding. Romania has the potential to become an attractive host for multinational clinical trials. This would bring financial benefits and offer patients access to cutting-edge treatments and therapies. However, to capitalize on these opportunities and become a competitive country in this field, Romania needs to devise a comprehensive strategy to attract global research investments, boosting the capabilities and financial sustainability of the healthcare system.

### 9. Local authorities' involvement and EU funds

To further enhance the healthcare capabilities of Romania, it is essential to equip a broader range of institutions and local actors with the skills and knowledge necessary to attract European funds. Promoting successful case studies of local authority involvement in securing and utilizing these funds can serve as an inspiring model and guide for others. By showcasing examples of good practice, we can encourage more widespread and effective participation in fund acquisition and management, ensuring a more equitable distribution of resources and benefits across the country, irrespective of the size or economic status of the city.
